# Interactions between Obstructive Sleep Apnea Syndrome Severity, Obesity, Sex Difference and Attention-Deficit/Hyperactivity Disorder on Health-Related Quality of Life: A Non-Interventional Prospective Observational Study

**DOI:** 10.3390/biomedicines10071576

**Published:** 2022-07-01

**Authors:** Yu-Hsuan Chen, Ming-Feng Wu, Chih-Yu Wen, Shih-Pei Chang, Ching-Yi Lin, Yi-Chan Chen, Ching-Cheng Lin, Hui-Chen Chen, Wei-Chang Huang, Kai-Ming Chang

**Affiliations:** 1 Department of Medical Laboratory Science and Biotechnology, Central Taiwan University of Science and Technology, Taichung 406, Taiwan; yhchen2@ctust.edu.tw (Y.-H.C.); heriknoha@vghtc.gov.tw (M.-F.W.); 2 Department of Internal Medicine, Division of Chest Medicine, Taichung Veterans General Hospital, Taichung 407, Taiwan; chingyii@vghtc.gov.tw (C.-Y.L.); aloneone0204@vghtc.gov.tw (Y.-C.C.); lucky_99@vghtc.gov.tw (C.-C.L.); huchen0328@vghtc.gov.tw (H.-C.C.); 3 Department of Electrical Engineering, National Chung Hsing University, Taichung 402, Taiwan; cwen@dragon.nchu.edu.tw; 4 Department of Physical Education, Central Taiwan University of Science and Technology, Taichung 406, Taiwan; spchang@ctust.edu.tw; 5 Department of Post-Baccalaureate Medicine, College of Medicine, National Chung Hsing University, Taichung 402, Taiwan; 6 Ph.D. Program in Translational Medicine, National Chung Hsing University, Taichung 402, Taiwan; 7 School of Medicine, Chung Shan Medical University, Taichung 402, Taiwan; 8 Department of Medical Technology, Jen-Teh Junior College of Medicine, Nursing and Management, Miaoli 350, Taiwan

**Keywords:** attention-deficit/hyperactivity disorder, health-related quality of life, obstructive sleep apnea syndrome

## Abstract

Obstructive sleep apnea syndrome (OSAS) severity, obesity, sex difference, and attention-deficit/hyperactivity disorder (ADHD) had a complex impact on health-related quality of life (HRQoL). However, the interactive effects among these features on HRQoL remained to be clarified. This study aimed to investigate the individual and interactive associations between the four characteristics of interest and HRQoL as determined by 36-Item Short Form Health Survey, Pittsburgh Sleep Quality Index (PSQI), and Epworth Sleepiness Scale (ESS). This non-interventional, prospective, observational study enrolled a total of 132 patients with suspected OSAS for analysis. While OSAS severity and ADHD detected by adult ADHD Self-Report Scale, termed as screened ADHD, interact with each other, all the four studied features were individually associated with HRQoL. After adjusting for potential physiological and polysomnographic confounders, screened ADHD was independently correlated with PSQI > 5 (OR = 4.126, 95% CI, 1.490–11.424), mental component score < 50 (OR = 5.873, 95% CI, 2.262–15.251) and ESS > 10 (OR = 3.648, 95% CI, 1.738–7.657). Our results show that ADHD detection is necessary and should be incorporated into clinical practice for OSAS management.

## 1. Introduction

Quality of life (QoL) is a complex, multifaceted concept encompassing the physical, psychological, and social domains that broadly indicate an individual’s well-being [1]. For what is related to healthcare, QoL has been applied to measure life concerns (e.g., health state, effects of illness, or satisfaction of the treatment), and is termed as “health-related quality of life (HRQoL)”. It includes the dimensions of physical function, mental state, social condition, and somatic perception [2]. For the assessment of HRQoL, a vast array of validated and reliable questionnaires are proposed, such as 36-Item Short Form Health Survey (SF-36), Pittsburgh Sleep Quality Index (PSQI), and Epworth Sleepiness Scale (ESS) [2,3,4]. 

Obstructive sleep apnea syndrome (OSAS) is characterized by recurrent partial or complete collapse of the upper airway, resulting in hypopnea or apnea [5,6]. It may lead to daytime sleepiness due to intermittent hypoxia and fragmented sleep. Moreover, it was reported to be an independent risk factor for cardiac, neurologic, and perioperative morbidities (i.e., difficult intubation, cardiac dysrhythmia, and longer hospital stay) that decrease the HRQoL due to effects on the patient’s physical and mental functions [7,8,9]. 

Obesity and sex difference, which were risk factors for OSAS [10,11,12,13], have also been associated with HRQoL [14,15]. It has shown that the poor sleep quality and metabolic as well as circadian disorders were correlated with obesity in young adults [16,17]. With regards to sex difference, a previous study found that females with chronic health conditions had worse physical and psychological domains of HRQoL compared to males [18]. In addition, women have been associated with a longer sleep latency and a 40% increased risk of developing insomnia compared to men [15]. 

Attention deficit hyperactivity disorder (ADHD) is a mental health disorder, common in childhood and often persisting into adulthood [19]. It is characterized by inattention, hyperactivity, and impulsive behavior that leads to unstable relationships and poor work as well as school performance [19]. Previous studies have shown that ADHD exhibited disabling effects on HRQoL, including poor self-esteem, substance abuse, driving, divorce, lost years of schooling, unemployment, underemployment, and as a risk for other disorders in both adults and children [20,21]. Furthermore, several studies have reported an association between ADHD and a variety of sleep disorders (e.g., OSAS) [22,23]. Meanwhile, Oğuztürk Ö et al. found that, compared to controls, patients with OSAS and ADHD had a higher anxiety score and worse HRQoL [24]. Taken together, these studies showed that HRQoL was an important clinical outcome measure. Meanwhile, although the interactive effects of these characteristics on HRQoL remained unclear, OSAS, obesity, sex difference, and ADHD all had complex impacts on HRQoL.

We hypothesized that the four features, including OSAS severity, obesity, sex difference, and ADHD, may have interactive effects on HRQoL as determined by SF-36, PSQI, and ESS. Therefore, this study aimed to explore the individual association between the four characteristics of interest and HRQoL, and to investigate whether the association was affected by other features.

## 2. Materials and Methods

### 2.1. Study Design, Setting, and Subject

This non-interventional, prospective, observational study was conducted at the sleep clinic of Taichung Veterans General Hospital (TCVGH), a medical center located at central Taiwan, between September 2015 and August 2017. The Institutional Review Board and Ethics Committee of TCVGH approved this study (approval number: CE15224A), and informed consent was obtained from all participants. 

### 2.2. Data Collection

At the sleep clinic, subjects were enrolled if they were aged ≥20 years and scheduled to undergo overnight polysomnography (PSG) for the suspicion of OSAS diagnosis as per sleep specialist’s comprehensive assessment of presenting symptoms and signs, medical history, and physical examination in a continuous, unbiased sampling manner. Those with a history of alcoholism or mental diseases other than ADHD or unable to answer the studied questionnaires were excluded from the study. 

At enrollment, physiological characteristics, including age, gender, body mass index (BMI), neck circumference, and waist circumference (WC) were recorded for each participant. Obesity was defined as a BMI ≥ 27 kg/m^2^ [25]. Meanwhile, all the eligible participants completed the adult ADHD Self-Report Scale (ASRS) and self-reported HRQoL assessments as detailed below and were scheduled to have a sleep test. The polysomnographic data were collected following overnight PSG.

### 2.3. Overnight PSG

As detailed elsewhere [26], OSAS was detected using overnight PSG (Compumedics, E-series, Melbourne, VIC, Australia). Hypopnea was defined as a ≥30% reduction in nasal pressure for ≥10 s with ≥4% oxygen desaturation, or a ≥50% reduction in nasal pressure for ≥10 s with ≥3% oxygen desaturation or arousal. Apnea was defined as a ≥90% reduction in airflow for ≥10 s. These events were manually scored by experienced technicians based on the recommendation of Task Force of the American Sleep Disorders Association 2007 [27]. The total number of apneas plus hypopneas divided by the total sleep time (hour) was defined as the apnea-hypopnea index (AHI). An AHI < 30 was defined as normal to moderate OSAS, and an AHI ≥ 30 was defined as severe OSAS.

### 2.4. Adult ASRS

The adult ASRS consists of 18 criteria outlined in the Diagnostic and Statistical Manual of Mental Disorders, Fourth Edition, and it was used to screen and diagnose ADHD, termed as “screened ADHD” in the present study. The ASRS consists of two subscales, inattention and hyperactivity, and each of which contains nine items. Each item is scored on a 5-point scale ranging from 0 (never) to 4 (very often). Participants with a sum score on either subscale of ≥17 were considered to have screened ADHD. Otherwise, non-screened ADHD was the case [28,29]. 

### 2.5. Self-Reported HRQoL

Self-reported HRQoL was assessed using the SF-36, Taiwan version (SF-36, Taiwan), PSQI, and ESS. The SF-36 consists of eight sections: vitality, physical functioning, bodily pain, general health perception, physical role function, emotional role function, social role function, and mental health. The scales are scored using a Likert method of summarized ratings, and the scores are weighted sums of the questions in each section. Each section is transformed into a 0–100 score. The SF-36 can also be divided into two components, the Physical Component Score (PCS) and the Mental Component Score (MCS). A lower score indicates more disable. In this study, a PCS or MCS score <50 was defined as worse HRQoL [30,31].

The PSQI is a standardized self-report questionnaire used to assess subjective sleep quality over the past month [32]. It consists of 19 items and seven clinically relevant components of sleep, including subjective sleep quality, sleep latency, sleep duration, sleep efficiency, sleep disturbances, use of sleep medication, and daytime dysfunction. The global PSQI score is calculated by adding the seven component scores, providing a total score ranging from 0 to 21. A score >5 indicates poor sleep quality [33].

The ESS is used to measure daytime sleepiness and ask the subject to rate his or her probability of falling asleep on a scale of increasing probability from 0 to 3 for eight different situations [34]. The scores of the eight questions are summed to obtain the total ESS score, with a score from 0–10 being considered normal, and a score >10 indicating the presence of excessive daytime sleepiness.

### 2.6. Outcome Assessment

As above-mentioned, the patients with worse HRQoL based on SF-36, PSQI, and ESS were defined as those with a PCS or MCS score <50, a score >5, and a score >10, respectively [30,31,33,34]. For the study purpose, it began with the exploration of the individual and interactive effects among OSAS severity, obesity, sex difference, and screened ADHD on SF-36, PSQI, and ESS. Next, these results would be adjusted by potential confounders, including physiological and polysomnographic parameters. 

### 2.7. Statistical Analysis

In this study, the enrolled participants were categorized into four binary groups: normal to moderate OSAS versus severe OSAS, female versus male, non-obese versus obese, and non-screened ADHD versus screened ADHD. A crossing matrix of three-way ANOVA was used to determine the individual and interactive effects among OSAS severity, obesity, sex difference, and screened ADHD on SF-36, PSQI, and ESS, respectively. A post hoc test was performed once the significant interaction or the main effect was met. Moreover, the univariate analysis was used to identify potential sleep and physiological parameters associated with HRQoL. Finally, combined the significant factors found in the three-way ANOVA analysis and those found in the univariate analysis, the independent variables associated with HRQoL were explored using the multiple logistic regression model. All data were expressed as mean and standard deviation (SD) for continuous variables or number (percentage) for categorical variables. The statistical analyses were performed using Predictive Analysis Software (PASW) version 18.0 (SPSS Inc., Chicago, IL, USA). Significance was set at *p* < 0.05.

## 3. Results

**Figure 1:**
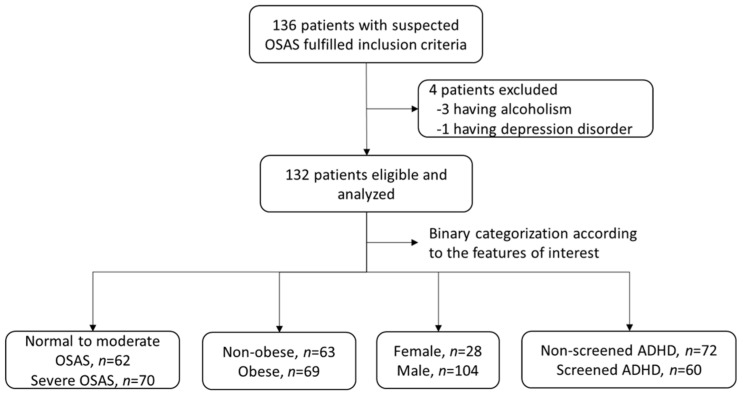
The patient enrollment flowchart.

Figure 1 shows that, a total of 132 subjects (104 (78.8%) males and 28 (21.2%) females) were enrolled in the final analysis. The mean ± SD of age, BMI, and AHI were 50.5 ± 13.4 years, 27.8 ± 4.2 kg/m^2^, and 39.5 ± 28.5, respectively (Table 1). Sixty (45.5%) of the participant had screened ADHD based on the results of the adult ASRS. The PSQI, PCS, MCS, and ESS scores were 9.2 ± 4.2, 47.4 ± 9.0, 43.2 ± 12.0, and 10.3 ± 5.3, respectively. 

**Figure 2:**
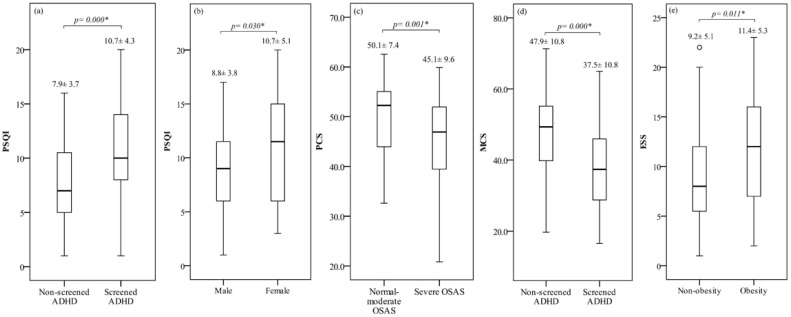
The significant results of HRQoL by the studied features with (**a**) PSQI by screened ADHD, (**b**) PSQI by sex difference, (**c**) PCS by OSAS severity, (**d**) MCS by screened ADHD, and (**e**) ESS by obesity. Abbreviations: ADHD, attention-deficit/hyperactivity disorder; ESS, Epworth Sleepiness Scale; MCS, mental component score; OSAS, obstructive sleep apnea syndrome; PCS, physical component score; PSQI, Pittsburgh Sleep Quality Index. * *p* < 0.05.

**Figure 3:**
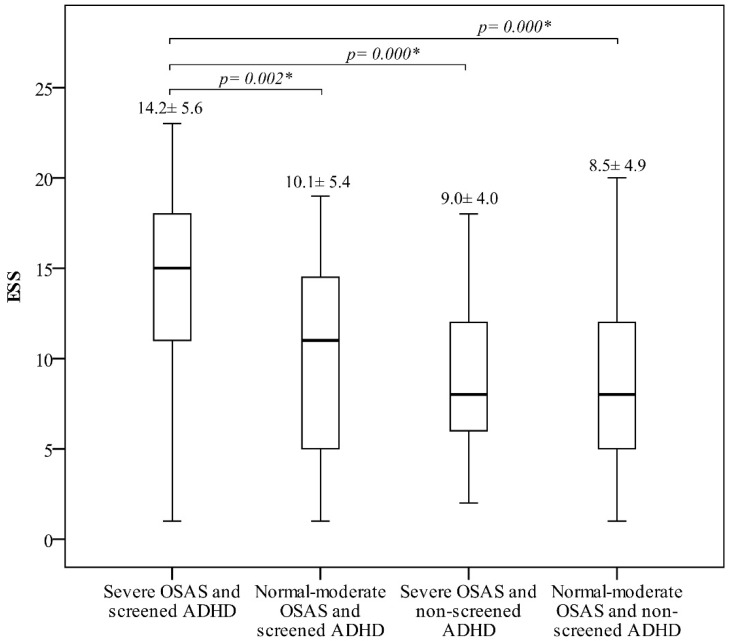
The ESS score for the interaction between OSAS severity and screened ADHD. * *p* < 0.05.

The three-way ANOVA analysis showed that screened ADHD and sex difference had a significant main effect on PSQI. Meanwhile, OSAS severity and screened ADHD had significant main effects on PCS and MCS, respectively. In addition, obesity exhibited a significant main effect while screened ADHD and OSAS severity presented an interactive effect with a significant simple main effect on ESS (Table 2). The details were provided in Figure 2a–e and Table S1. Based on the finding that an interactive effect on ESS existed between ADHD and OSAS severity, we further analyzed and found that, patients with severe OSAS and screened ADHD had the highest ESS score as compared to other study groups (Figure 3).

Since physiological or sleep variables may potentially contribute to HRQoL, we analyzed the individual effect between these parameters and HRQoL. The results showed that age and WC were associated with MCS (*p* = 0.001) and PCS (*p* = 0.034), respectively. Moreover, the average oxygen saturation during sleep correlated to ESS (*p* = 0.047) while sleep stages had a relationship with PSQI (stage N1, *p* = 0.009; stage N3, *p* < 0.001), PCS (rapid eye movement stage, *p* = 0.031), and MCS (stage N1, *p* = 0.001; stage N2, *p* = 0.002) (Table 3). Together with those significant effects found in the three-way ANOVA analysis, the multiple logistic regression analysis showed that, patients with screened ADHD were independently more likely to have PSQI > 5, MCS < 50, and ESS > 10 when compared to those with non-screened ADHD while OSAS severity, obesity, and sex difference had no significant effects on PSQI, MCS, PCS, or ESS (Table 4).

## 4. Discussion

We found that, although the four studied features, including OSAS severity, obesity, sex difference and screened ADHD, were associated with HRQoL, as determined by SF-36, PSQI and ESS in the three-way ANOVA analysis, particularly an interactive effect between OSAS severity and screened ADHD on ESS score, only patients with screened ADHD were more likely to have worse HRQoL, comparing to those with non-screened ADHD after adjusting for potential physiological and sleep variables in the multiple logistic regression analysis.

Similar to our findings that screened ADHD was independently associated with ESS score > 10 in patients with suspected OSAS, Oğuztürk Ö et al. have shown that, when compared to OSAS patients without ADHD, those with ADHD had a higher anxiety score, a poorer PCS of SF-36, and a higher ESS score [24]. This could at least partly explain why severe OSAS patients presented with a lower ESS score and vice versa encountered in clinical practice.

In this study, for patients with suspected OSAS, the relationship between OSAS severity and ESS score was affected by screened ADHD in addition to the correlations between the studied features and the tested questionnaires for HRQoL in the three-way ANOVA analysis. We also have reported that OSAS severity and sex difference had an interactive effect on serum interleukin-6 level, cardiovascular biomarker, in patients suspected to have a diagnosis of OSAS [26]. Moreover, one previous review article showed that ADHD and sleep disorders may interact and share a common underlying neurological etiology [35]. Taken together, a comprehensive evaluation of patient characteristics, concurrent mental disorders, blood inflammatory biomarkers, and HRQoL is recommended for the management of patients with OSAS.

Together with our findings that OSAS severity was associated with a worse HRQoL as determined by PCS and is interactive with screened ADHD on ESS score, evidence has shown that OSAS decreased HRQoL in different dimensions [7,8,9]. This could raise physician’s awareness that OSAS has a great impact on patient’s HRQoL and the access to diagnosis. Thus, treatment for OSAS should be facilitated as there is a long waiting list for PSG to diagnose and treat this disorder worldwide [36].

The strengths of this study include that a three-way ANOVA analysis was used to examine the association between the four features of interest and the three studied questionnaires related to HRQoL. This made it easier to clarify the complex relationship between these features and HRQoL. Furthermore, we used multiple logistic regression model to incorporate other potential confounders into our analysis, making our results solider for interpretation. This compensates several limitations of our study, including an inherent limitation of studies on OSAS that our results are subject to selection bias toward middle-aged male adults, which may make the results not generalizable to other populations. Moreover, the diagnosis of ADHD was made by adult ASRS, thus termed as screened ADHD, making it less accurate in the detection of this mental health disorder. Meanwhile, the sample size had not been calculated, making the power of the study may be sub-optimal.

ADHD is a common mental disorder with a prevalence of 3–16% in children, enduring through adulthood in about 60%–80% of cases [24,35]. Moreover, in our study, as high as 45.5% of participants had screened ADHD detected by adult ASRS. Meanwhile, patients with screened ADHD were independently associated with a worse HRQoL as compared to those with non-screened ADHD. In sum, apart from the common patient characteristics that would affect HRQoL, the assessment of ADHD could not be neglected and should be incorporated into the clinical practice for the management of OSAS. Future studies are required to clarify the impact of mentality on HRQoL in such population by including more patients with different kinds of mental health disorders.

## 5. Conclusions

We found an independent correlation between screened ADHD and ESS score > 10 in patients with suspected OSAS. Meanwhile, OSAS severity, obesity, and sex difference also had individual associations with HRQoL in such population. This information made the detection of ADHD necessary in the management of OSAS.

## Figures and Tables

**Table 1:** The baseline characteristics of the enrolled subjects (*n* = 137).

	Variable	Mean ± SD/*n* (%)
Demography	Number of male	104 (78.8%)
	Age (years)	50.5 ± 13.4
	BMI (kg/m^2^)	27.8 ± 4.2
	Number of obesity	69 (52.3%)
	NC (cm)	39.0 ± 3.8
	WC (cm)	96.0 ± 11.6
Polysomnography	AHI	39.5 ± 28.5
	Number of severe OSAS	70 (53.0%)
	SpO_2nadir_ (%)	79.2 ± 9.7
	SpO_2mean_ (%)	93.1 ± 3.8
	Sleep stage N1 (%)	26.7 ± 19.1
	Sleep stage N2 (%)	52.4 ± 16.4
	Sleep stage N3 (%)	8.7 ± 9.5
	Sleep stage REM (%)	11.5 ± 6.5
	TRT (min)	367.1 ± 14.7
	TST (min)	317.1 ± 37.7
	SE (%)	86.3 ± 9.7
Questionnaire	Number of screened ADHD	60 (45.5%)
	PSQI	9.2 ± 4.2
	Number of PSQI > 5	104 (78.8%)
	PCS	47.4 ± 9.0
	Number of PCS < 50	73 (55.3%)
	MCS	43.2 ± 12.0
	Number of MCS < 50	89 (67.4%)
	ESS	10.3 ± 5.3
	Number of ESS > 10	62 (47.0%)

Abbreviations: ADHD, attention-deficit/hyperactivity disorder; AHI, apnea-hypopnea index; BMI, body mass index; ESS, Epworth Sleepiness Scale; MCS, mental component score; NC, neck circumference; OSAS, obstructive sleep apnea syndrome; PCS, physical component score; PSQI, Pittsburgh Sleep Quality Index; REM, rapid eye movement; SD, standard deviation; SE, sleep efficiency; SpO2_nadir_, the lowest oxygen saturation during sleep; SpO2_mean_, the average oxygen saturation during sleep; TRT, total recording time; TST, total sleep time; WC, waist circumference.

**Table 2:** The crossing matrix for self-reported HRQoL in the three-way ANOVA analysis.

Three-Way Factor	PSQI	PCS	MCS	ESS
A, B, S	A, S	-	A	A, B
A, B, O	A	O	A	A, O
A, O, S	A, S	-	A	A∩O
B, O, S	-	O	-	-
Significant factor	A, S	O	A	B, A∩O

Abbreviations: A, attention-deficit/hyperactivity disorder; B, obesity; HRQoL, health-related quality of life; O, obstructive sleep apnea syndrome severity; S, sex difference; also see Table 1. ∩: interaction.

**Table 3:** The univariate analysis of potentially associated factors for self-reported HRQoL.

	PSQI	PCS	MCS	ESS
Age (year)	0.231	0.1130	0.001 *	0.544
NC (cm)	0.280	0.067	0.406	0.588
WC (cm)	0.114	0.034 *	0.128	0.579
SpO_2nadir_ (%)	0.198	0.616	0.941	0.065
SpO_2mean_ (%)	0.916	0.288	0.833	0.047 *
Stage N1(%)	0.009 *	0.224	0.001 *	0.814
Stage N2 (%)	0.127	0.482	0.002 *	0.653
Stage N3 (%)	0.000 *	0.568	0.067	0.839
REM (%)	0.472	0.031 *	0.561	0.772
SE (%)	0.553	0.685	0.134	0.074

Abbreviations: see Table 1 and Table 2. The difference was determined by independent *t*-test. * *p* < 0.05.

**Table 4:** The significant factors for self-reported HRQoL in the multiple logistic regression analysis.

Main Effect			
Outcome	Factors	Odds Ratio (95% CI)	*p*-Value
PSQI > 5	Screened ADHD vs. non-screened ADHD	4.126 (1.490–11.423)	0.006 *
	Stage N1 (per 1% increase)	0.985 (0.962–1.008)	0.208
	Stage N3 (per 1% increase)	1.077 (1.000–1.160)	0.049 *
PSQI > 5	Female vs. male	0.674 (0.204–2.224)	0.518
	Stage N1 (per 1% increase)	0.982 (0.959–1.006)	0.137
	Stage N3 (per 1% increase)	1.072 (0.998–1.152)	0.056
PCS < 50	Severe OSAS vs. normal-moderate OSAS	1.948 (0.842–4.508)	0.119
	WC (per 1cm increase)	1.016 (0.979–1.054)	0.411
	REM (per 1% increase)	0.949 (0.897–1.005)	0.075
MCS < 50	Screened ADHD vs. non-screened ADHD	5.873 (2.262–15.251)	0.000 *
	Age (per 1 year increase)	0.971 (0.940–1.003)	0.078
	Stage N1 (per 1% increase)	0.974 (0.943–1.006)	0.105
	Stage N2 (per 1% increase)	1.010 (0.974–1.047)	0.589
ESS > 10	Obesity vs. non-obesity	1.662 (0.799–3.460)	0.174
	SpO_2mean_ (per 1% increase)	0.922 (0.830–1.025)	0.132
**Simple main effect**			
Outcome	Factors	Odds ratio (95% CI)	*p*-value
ESS > 10	Severe OSAS vs. normal-moderate OSAS	1.215 (0.518–2.846)	0.655
	Screened ADHD vs. non-screened ADHD	3.648 (1.738–7.657)	0.001 *
	SpO_2mean_ (per 1% increase)	0.909 (0.808–1.024)	0.116

Abbreviations: CI, confidence interval; also see Table 1 and Table 2. * *p* < 0.05.

## Data Availability

Data is contained within the article and Supplementary materials.

## Supplementary Materials

The following supporting information can be downloaded at: https://www.mdpi.com/article/10.3390/biomedicines10071576/s1, Table S1: The details of the crossing matrix for self-reported HRQoL in the three-way ANOVA analysis.
