# Supplementary material for: Interactions between Obstructive Sleep Apnea Syndrome Severity, Obesity, Sex Difference and Attention-Deficit/Hyperactivity Disorder on Health-Related Quality of Life: A Non-Interventional Prospective Observational Study
Source: Biomedicines. 2022 Jul 1;10(7):1576. doi: 10.3390/biomedicines10071576 (PMC9313041; doi:10.3390/biomedicines10071576)
Supplement: Supplementary file 1 [file biomedicines-10-01576-s001.zip › biomedicines-1766533-supplementary.pdf]

Table S1. The details of the crossing matrix for self-reported HRQoL in the three-way ANOVA analysis.

Three-	PSQI				PCS				MCS				ESS			
way	SS	MS	F	p-value	SS	MS	F	p-value	SS	MS	F	p-value	SS	MS	F	p-value
A	148.42	148.42	9.770	0.002*	38.09	38.09	0.459	0.500	1301.1	1301.1	11.211	0.011*	277.95	277.95	11.053	0.001*
B	40.92	40.92	2.694	0.103	3.71	3.71	0.045	0.833	6.25	6.25	0.054	0817	159.04	159.04	6.325	0.013*
S	98.97	98.97	6.515	0.012*	39.65	39.65	0.477	0.491	3.45	3.45	0.030	0.863	1.02	1.02	0.041	0.841
A× B	16.60	16.60	1.092	0.298	39.47	39.47	0.475	0.492	62.76	62.76	0.541	0.463	13.34	13.34	0.530	0.468
A× S	2.19	2.19	0.144	0.705	25.41	25.41	0.306	0.581	404.28	404.28	3.484	0.064	9.87	9.87	0.396	0.530
S× B	18.55	18.55	1.221	0.271	7.72	7.72	0.093	0.761	296.97	296.97	2.559	0.112	10.90	10.90	0.434	0.511
A× B× S	15.39	15.39	1.013	0.316	0.02	0.02	0.000	0.987	5.595	5.595	0.048	0.827	4.97	4.97	0.198	0.657
SSE	1883.8	15.2			10230	83.1			14390	116.0			3093.1	25.1		
SST	2274.3				10570				18743				3620.1			
A	233.70	233.70	14.656	0.000*	243.76	243.76	3.261	0.073	2898.2	2898.2	24.259	0.000*	292.07	292.07	12.175	0.001*
B	22.45	22.45	1.407	0.238	97.27	97.27	1.301	0.256	5.17	5.17	0.043	0.835	62.75	62.75	2.616	0.108
O	13.53	13.53	0.848	0.359	1069.9	1069.9	14.312	0.000*	283.7	283.7	2.375	0.126	82.87	82.87	3.454	0.108
A× B	6.42	6.42	0.402	0.527	176.15	176.15	2.356	0.127	15.38	15.38	0.129	0.720	6.27	6.27	0.261	0.610
A× O	0.554	0.554	0.035	0.853	83.44	83.44	1.116	0.293	2.76	2.76	0.023	0.879	97.68	97.68	4.072	0.046*
B× O	15.14	15.14	0.949	0.332	2.79	2.79	0.037	0.847	22.08	22.08	0.185	0.668	9.71	9.71	0.405	0.526
A× B× O	7.68	7.68	0.481	0.489	61.87	61.87	0.828	0.365	2.51	2.51	0.021	0.885	1.17	1.17	0.049	0.825
SSE	1978.7	16.0			9269.1	74.8			14813				2950.8	123.0		
SST	2274.3				10570				18743				3620.1			

A	133.83	133.3	8.616	0.004*	10.74	10.74	0.146	0.703	1165.6	1165.6	10.136	0.002*	201.88	201.88	8.423	0.004*
S	70.38	70.39	4.532	0.035*	60.34	60.34	0.821	0.367	0.235	0.235	0.002	0.964	0.646	0.646	0.027	0.870
O	0.42	0.42	0.027	0.870	296.13	296.13	4.027	0047*	67.78	67.78	0.589	0.444	70.48	70.48	2.940	0.089
A× S	1.84	1.84	0.118	0.732	61.98	61.98	0.843	0.360	300.79	300.79	2.616	0.108	7.33	7.33	0.306	0.581
A× O	3.44	3.44	0.222	0.639	44.18	44.18	0.601	0.440	9.12	9.12	0.079	0.779	17.31	17.31	0.72	0.397
S× O	1.65	1.65	0.106	0.745	121.49	121.49	1.652	0.201	134.52	134.52	1.170	0.282	2.73	2.73	0.114	0.736
A× S× O	8.58	8.58	0.553	0.459	164.92	164.92	2.243	0.137	19.52	19.52	0.170	0.681	57.62	57.62	2.404	0.124
SSE	1926.0	124.0			9118.2	73.5			14259	115.0			2948.2	24.0		
SST	2274.3				10570				18743				3620.1			
B	54.76	54.76	3.192	0.076	21.06	21.06	0.276	0.600	108.40	108.40	0.762	0.384	94.0	94.0	3.393	0.068
S	39.03	39.03	2.275	0.134	111.6	111.6	1.457	0.230	9.07	9.07	0.064	0.801	0.2	0.2	0.009	0.926
O	22.85	22.85	1.332	0.251	389.23	389.23	5.106	0.026*	217.09	217.09	1.527	0.219	9.0	9.0	0.326	0.569
B× S	24.88	24.88	1.450	0.231	0.26	0.26	0.003	0.954	295.43	295.43	2.077	0.152	11.9	11.9	0.430	0.513
B× O	1.09	1.09	0.063	0.802	0.46	0.46	0.006	0.938	104.77	104.77	0.737	0.392	0.1	0.1	0.002	0.963
S× O	13.37	13.37	0.779	0.379	64.47	64.47	0.846	0.360	32.28	32.28	2.262	0.610	9.0	9.0	0.326	0.569
B× S× O	10.43	10.43	0.608	0.437	4.98	4.98	0.065	0.799	81.45	81.45	0.573	0.451	1.2	1.2	0.007	0.933
SSE	2127.2	17.2			9452.5	76.2			17634	142.2			3406.3	27.7		
SST	2274.3				10570				18743				3620.1			
Significant effect	A; S				O				A				B; A∩O			

Abbreviations: A, attention-deficit/hyperactivity disorder; B, obesity; ESS, Epworth Sleepiness Scale; F, F-value; HRQoL, health-related quality of life; MCS, mental component score; MS, mean square; O, obstructive sleep apnea syndrome severity; PCS, physical component score; PSQI, Pittsburgh Sleep Quality Index; S, sex difference; SS, total sum of square of deviation from mean; SSE: the sum of squared estimate of errors; SST: the sum of squares total.

* $p < 0.05$.

∩: interaction.
